# Pancreatic ductal adenocarcinoma: biological hallmarks, current status, and future perspectives of combined modality treatment approaches

**DOI:** 10.1186/s13014-019-1345-6

**Published:** 2019-08-08

**Authors:** Michael Orth, Philipp Metzger, Sabine Gerum, Julia Mayerle, Günter Schneider, Claus Belka, Maximilian Schnurr, Kirsten Lauber

**Affiliations:** 1 Department of Radiation Oncology, University Hospital, LMU Munich, Munich, Germany; 2 German Cancer Consortium (DKTK), LMU Munich, 81377 Munich, Germany; 3 German Cancer Research Center (DKFZ), Heidelberg, Germany; 4 Center of Integrated Protein Science Munich (CIPSM) and Division of Clinical Pharmacology, University Hospital, LMU Munich, Munich, Germany; 5 Department of Internal Medicine II, University Hospital, LMU Munich, Munich, Germany; 6 Department of Internal Medicine II, Klinikum rechts der Isar, Technical University Munich, Munich, Germany

## Abstract

Pancreatic ductal adenocarcinoma (PDAC) is a highly devastating disease with poor prognosis and rising incidence. Late detection and a particularly aggressive biology are the major challenges which determine therapeutic failure. In this review, we present the current status and the recent advances in PDAC treatment together with the biological and immunological hallmarks of this cancer entity. On this basis, we discuss new concepts combining distinct treatment modalities in order to improve therapeutic efficacy and clinical outcome – with a specific focus on protocols involving radio(chemo)therapeutic approaches.

## Introduction

Pancreatic ductal adenocarcinoma (PDAC) is the most prevalent neoplastic disease of the pancreas accounting for more than 90% of all pancreatic malignancies [1]. To date, PDAC is the fourth most frequent cause of cancer-related deaths worldwide with a 5-year overall survival of less than 8% [2]. The incidence of PDAC is expected to rise further in the future, and projections indicate a more than two-fold increase in the number of cases within the next ten years, both in terms of new diagnoses as well as in terms of PDAC-related deaths in the U.S. as well as in European countries ([3, 4], www.cancerresearchuk.org/health-professional/cancer-statistics/statistics-by-cancer-type/pancreatic-cancer#heading-Zero). A particular reason for this – apart from the general aging of our society – is the evident implication of obesity and type 2 diabetes, two emerging public health challenges, in PDAC etiology [5–7]. Life style habits, including alcohol and tobacco abuse, which are well-known to increase the risk for several other types of cancer, such as lung cancer and squamous cell carcinomas of the head and neck region [8–10], also appear to be involved in PDAC development [11–15]. Finally, for a subgroup of approximately 5-6% of all PDAC patients, genetic predispositions, such as germline mutations in the genes *BRCA1/2, ATM, MLH1, TP53,* or *CDKN2A*, represent further risk factors [16–18].

### Current treatment standards and recent advances in PDAC chemo- and/or radiotherapy

Efficacy and outcome of PDAC treatment are largely determined by the stage of disease at the time of diagnosis. Surgical resection followed by adjuvant chemotherapy is the only possibly curative therapy available, yet only 10-20% of PDAC patients present with resectable PDAC stages, while the residual 80-90% show locally advanced, non-resectable stages or – in the majority – distant metastases [19, 20]. Systemic chemotherapy is commonly employed as first-line treatment in patients with non-resectable or borderline-resectable tumors. This encompasses nucleoside analogues, including gemcitabine and capecitabine, or the pyrimidine analogue 5-fluorouracil (5-FU) in monotherapy settings or in combination with other treatment modalities, such as radiotherapy, respectively [20–22]. FOLFIRINOX, a poly-chemotherapeutic regimen composed of folinic acid, 5-FU, irinotecan, and oxaliplatin, has been reported to nearly double median survival in the metastasized stage as compared to gemcitabine alone [23], and the combination of gemcitabine and a nanoparticle albumin-bound paclitaxel (nab-paclitaxel) has also been shown to significantly improve overall survival [24]. However, these protocols are associated with relevantly higher toxicity, thus often preventing their application in elderly patients and/or patients with poor performance status, but overall quality of life was reported to increase [25].

Radio(chemo)therapy has been rather infrequently adopted for the treatment of PDAC, since the majority of patients suffer from disseminated stages in which local treatment procedures are of secondary importance [26]. Nevertheless, neoadjuvant radiotherapy has the potential to improve PDAC resectability in locally advanced or primarily inoperable/borderline-operable patients, and its beneficial effects on local tumor control are well documented [27, 28]. Compared to other cancer entities, PDAC tumors exhibit a rather high degree of radioresistance – a characteristic which is currently addressed by combining PDAC radiotherapy with radiosensitizing agents, including gemcitabine, capecitabine, or 5-FU, respectively [28, 29]. According to the guidelines of the National Comprehensive Cancer Network (NCCN), the use of radio(chemo)therapy is recommended for PDAC patients with borderline-resectable tumors, and several regimens involving capecitabine, gemcitabine, or 5-FU have been clinically implemented [29, 30]. The advances of modern external beam radiation techniques, including image-guided radiation therapy (IGRT), stereotactic body radiation therapy (SBRT), and ablative radiation therapy, as well as the combination with novel chemotherapeutic protocols have clearly widened the spectrum of radiotherapeutic options [27, 31, 32].

Expecting increased toxicities when combining more aggressive treatment approaches, sequential application is currently being evaluated in the randomized phase III CONKO-007 trial for PDAC patients with borderline-resectable, non-metastatic disease (NCT01827553). Preliminary results from an interims analysis document a promising outcome with higher rates of resectability, confirming previous phase II findings [27, 30, 33]. As the performance of systemic therapies gradually improves, local tumor control moves back into the focus of interest, both with respect to symptom control as well as with respect to quality of life. In consequence, the importance of local radiotherapy for the treatment of PDAC patients is constantly growing. SBRT is a highly conformal radiation technique which is employed to deliver high doses in a small number of fractions. Due to its steep dose gradients around the target volume, SBRT efficiently spares adjacent organs at risk resulting in relevantly lower toxicity. In several studies, SBRT achieved significant improvements in pain control paralleled by increased local tumor control [34]. Hence, SBRT can be seen as an effective and safe therapeutic option, and its use in multimodality treatment concepts and/or in palliative settings is considered more and more frequently.

In several other cancer entities, e.g. in melanoma and lung cancer, the implementation of immunotherapeutic approaches, specifically immune checkpoint inhibition, has proven compelling success [35–38]. Yet, at least so far, treatment efficacy in PDAC has been rather limited [35, 39], and checkpoint inhibition has only received approval for the small subset of PDAC tumors with high microsatellite instability (1-2% of all cases) [40, 41]. This may be due to the strongly immunosuppressive, desmoplastic PDAC microenvironment, the relatively low mutational burden (resulting in a low number of neo-antigens), as well as other biological and/or immunological hallmarks of PDAC which are discussed in this review [42].

### Biological and immunological hallmarks of PDAC

#### Tumor plasticity and heterogeneity

**Fig. 1:**
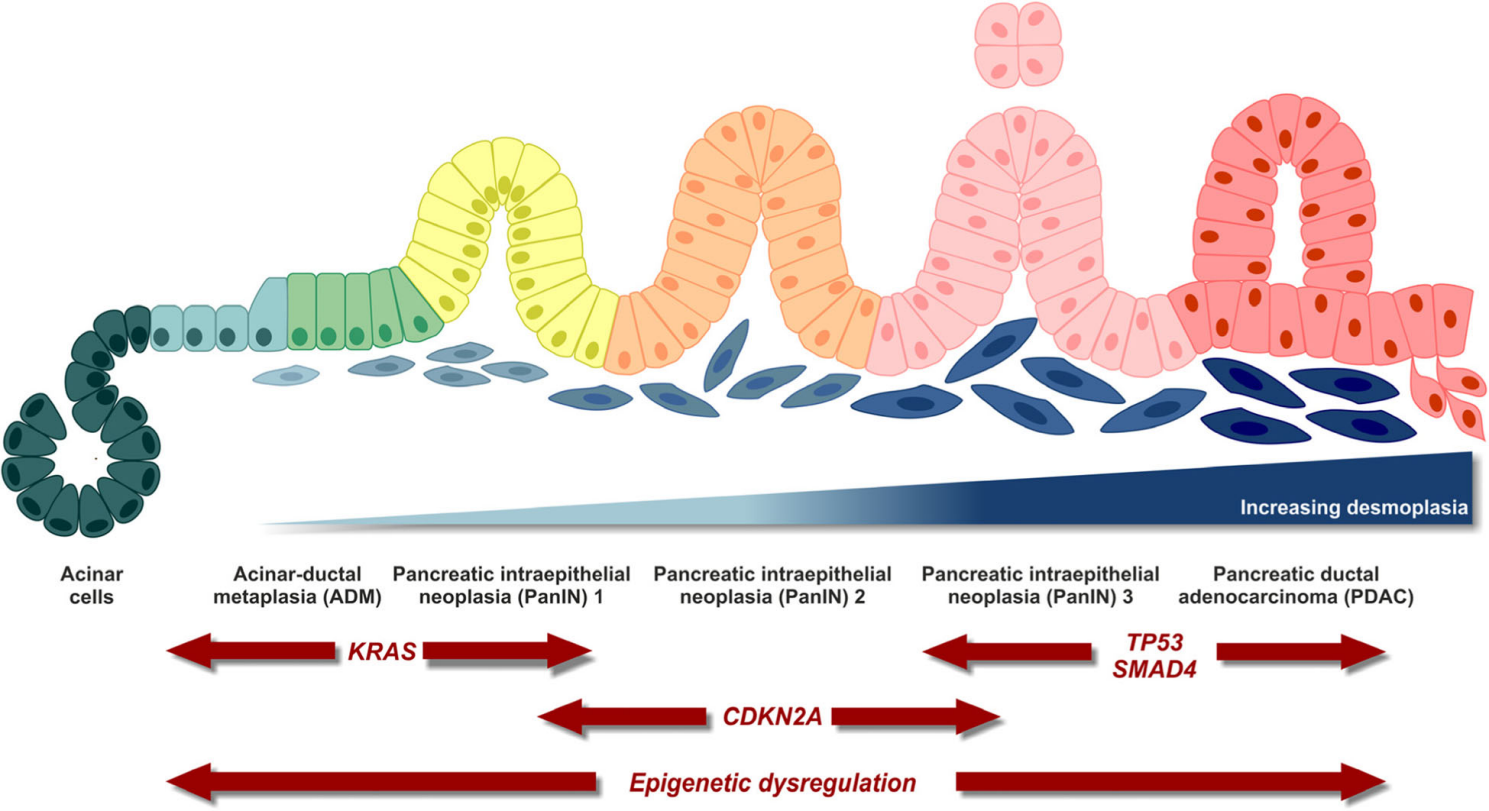
Multi-step PDAC carcinogenesis. Modified from [47].

The pancreas contains cells of exocrine (acinar), epithelial (ductal), and endocrine (α, β, δ, ε) origin among which acinar cells are well known for their high degree of plasticity. This plasticity is considered to drive pancreas homeostasis and regeneration, as – in contrast to other organs of the gastrointestinal tract – the pancreas seems to lack a defined stem cell compartment [43]. In a process called acinar-to-ductal metaplasia (ADM), acinar cells transdifferentiate to more epithelial (ductal-like) phenotypes when experiencing certain macro- and microenvironmental stimuli, e.g. tissue damage, inflammatory, or stress conditions [44, 45]. During ADM, acinar cells acquire ‘progenitor cell-like’ characteristics which render them more susceptible to pro-oncogenic hits, such as activating mutations in the proto-oncogene *KRAS*, eventually transforming them into pancreatic intra-epithelial neoplasias (PanINs). This transformation is generally considered as the initial step in PDAC development followed by sequential progression involving genetic hits in several tumor suppressor genes [46] (Fig. 1).

In order to examine the mutational and transcriptional landscape of PDAC, a number of next generation sequencing approaches were initiated in the last years [48–51]. In conjunction, these studies showed that the gene encoding the proto-oncogenic GTPase *KRAS* as well as several tumor suppressor genes, including *tumor suppressor protein 53* (*TP53)*, *cyclin-dependent kinase inhibitor 2A* (*CDKN2A),* and *mothers against decapentaplegic homologue 4 (SMAD4),* exhibit the most frequent alterations and/or mutations in PDAC [49]. For instance, *KRAS* was not only found to be mutated in most PDAC tumors (> 90%), its mutant alleles were additionally amplified in a subgroup of samples, resulting in acceleration of their tumor-promoting potential [52]. Furthermore, *RAC-beta serine/threonine-protein kinase (AKT2)* is frequently overexpressed, and the activity of its upstream regulator *phosphoinositide 3-kinase (PI3K)* is often elevated in PDAC leading to increased tumor cell survival [53, 54]. Apart from these key mutations, several more uncommon alterations, such as germline mutations in DNA damage repair genes (e.g. *breast cancer early onset genes 1/2 (BRCA1/2)*, *partner and localizer of BRCA2 (PALB2),* and *ataxia telangiectasia mutated protein* serine/threonine kinase *ATM),* or somatic mutations in DNA mismatch repair regulator genes leading to increased microsatellite instability have been found in certain subsets of patients [55]. Of note, the transcriptomic landscape of PDAC is not entirely governed by genetic alterations. Integrated epigenetic regulatory circuits comprising chromatin-based mechanisms, such as DNA methylation and histone post-translational modification, as well as regulation by non-coding RNAs are also largely distorted in PDAC. In this regard, key tumor suppressor genes have been described to be repressed, and oncogenes upregulated due to epigenetic alterations [56]. Furthermore, epigenetic (re-)programing is fundamentally linked to tumor progression and metastasis formation [57, 58], and the epigenetic landscapes of human PDAC subtypes differ substantially [59].

**Fig. 2:**
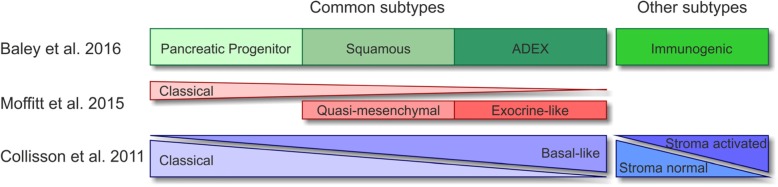
Molecular classifications of PDAC. Modified from [63].

PDAC is a highly heterogenic disease, and various attempts have been undertaken to define distinct subtypes with the aim of stratifying patients towards personalized treatment strategies [49, 50, 60–62]. Currently available transcriptome-based classifications were extracted via unsupervised clustering methods and differ in the numbers of subtypes identified. Nevertheless, all share common subtypes, including a classical/canonical subtype hallmarked by epithelial-like gene expression, and a quasi-mesenchymal/basal-like subtype characterized by a more mesenchymal gene expression pattern and poorer prognosis (Fig. 2). These subtypes meanwhile can be stratified by immunohistochemistry using *hepatocyte nuclear factor 1A* (*HNF1A*) and *cytokeratin-81 (KRT81)* as markers [64]. Furthermore, subtypes related to exocrine pancreas function have been described as well as subtypes with expression signatures of immune cell-related genes [50, 61, 62]. Although to date there is still no consensus classification which would be the prerequisite for clinical application, retrospective as well as prospective analyses have shown that subtype-based stratification has the potential for genomics-driven precision medicine [64, 65]. The PDAC subtypes obviously stem from inter-tumoral heterogeneity. Yet, intra-tumoral heterogeneity needs to be considered as well, and tumor cell plasticity might render these classifications dynamic, especially upon therapeutic intervention.

#### Desmoplastic, hypoxic, immunosuppressive microenvironment

**Fig. 3:**
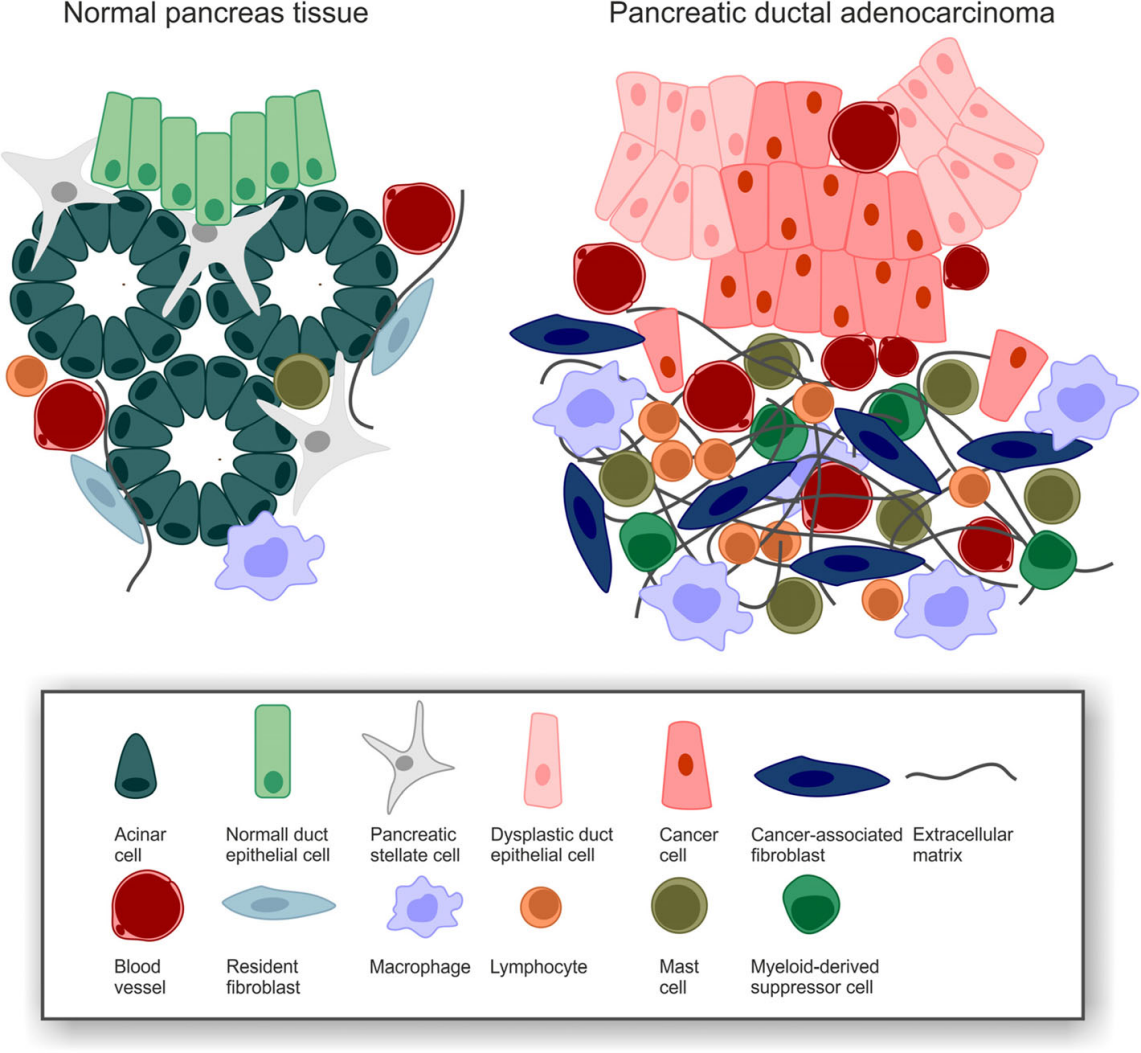
PDAC desmoplasia. Modified from [66].

A crucial hallmark of PDAC is the existence of extensive desmoplastic stroma which can constitute up to 90% of the tumor volume and is commonly considered to originate from cancer-associated fibroblasts (CAFs) [42] (Fig. 3). Distinct subtypes of CAFs with either myofibroblastic or inflammatory phenotypes have been identified [67, 68], and the major source of CAFs appear to be pancreatic stellate cells which upon activation, e.g. by injury or chronic inflammation, start depositing huge amounts of extra cellular matrix, including laminins, fibronectins, collagens, and hyaluronan [69–72]. Interestingly, expression of *focal adhesion kinase 1* (*FAK1*) in PDAC cells has recently been reported to be decisive for this process as pharmacological targeting of *FAK1* interfered with the formation of desmoplasia, thus offering a potential target for therapeutic intervention [73]. Hypoxia is another key feature of the PDAC microenvironment, and it is closely interlinked with desmoplasia. It originates from desmoplasia-associated hypovascularization and *vice versa* favors desmoplastic progression by activating pancreatic stellate cells [74–76]. PDAC hypoxia and desmoplasia, which are observed in clinical samples as well as in genetically engineered mouse models, seem to represent barriers to T cell infiltration – intriguingly both for effector as well as regulatory T cells – and T cell activation [77–79]. Moreover, hypoxia and desmoplasia are accompanied by a strong accumulation of myeloid cells [80, 81]. Macrophages that are recruited adopt an immunosuppressive, pro-angiogenic M2-like state, block CD4^+^ T cell entry into the PDAC microenvironment, support PDAC progression, and thus are a marker of negative clinical prognosis [76, 82, 83]. Systemic frequencies of monocytes and granulocytes are elevated in PDAC patients, and due to their pathological activation and immunosuppressive function they are classified as monocytic or polymorphonuclear myeloid-derived suppressor cells (MDSCs), respectively. Both populations are potent suppressors of T cell function and inhibit anti-tumor immune responses [84, 85]. Recently, the *CXCL-1/CXCR2*-axis has been shown to be crucially involved in intra-tumoral recruitment of MDSCs, suppressing CD8^+^ T cell infiltration and function as well as compromising responsiveness to immunotherapy [86]. Apart from these innate immune cell subpopulations, immunosuppressive T and B cell subpopulations, including regulatory T cells, γδ T cells, and regulatory B cells, have been described in the PDAC microenvironment. They do not only block activation but also infiltration of effector T cells resulting in low intra-tumoral CD8^+^ T cell frequencies [87–89]. These effector T cells appear to be antigen-experienced, but tumor antigen recognition and/or T cell activation seem to be disturbed [90]. However, the intra-tumoral T cell repertoire shows enrichment in distinct T cell receptors, suggesting that in principle PDAC tumors are sites of local T cell expansion [91].

On the cytokine level, the PDAC microenvironment represents a comparable degree of complexity. Nevertheless, the dominating cytokines seem to be *transforming growth factor beta (TGF-β), interleukin (IL-) 6, IL-8, IL-10, IL-35, granulocyte macrophage colony-stimulating factor (GM-CSF), CC-chemokine ligand 2 (CCL-2), CXC-chemokine ligand 1 (CXCL-1), and CXCL-13*. In complex networks they orchestrate the recruitment and education of innate and adaptive immune cells as well as their crosstalk with tumor cells, CAFs, and other cells in the PDAC microenvironment, culminating in the desmoplastic, immunosuppressive milieu that has been described above [92–94].

#### Metastasis formation

**Fig. 4:**
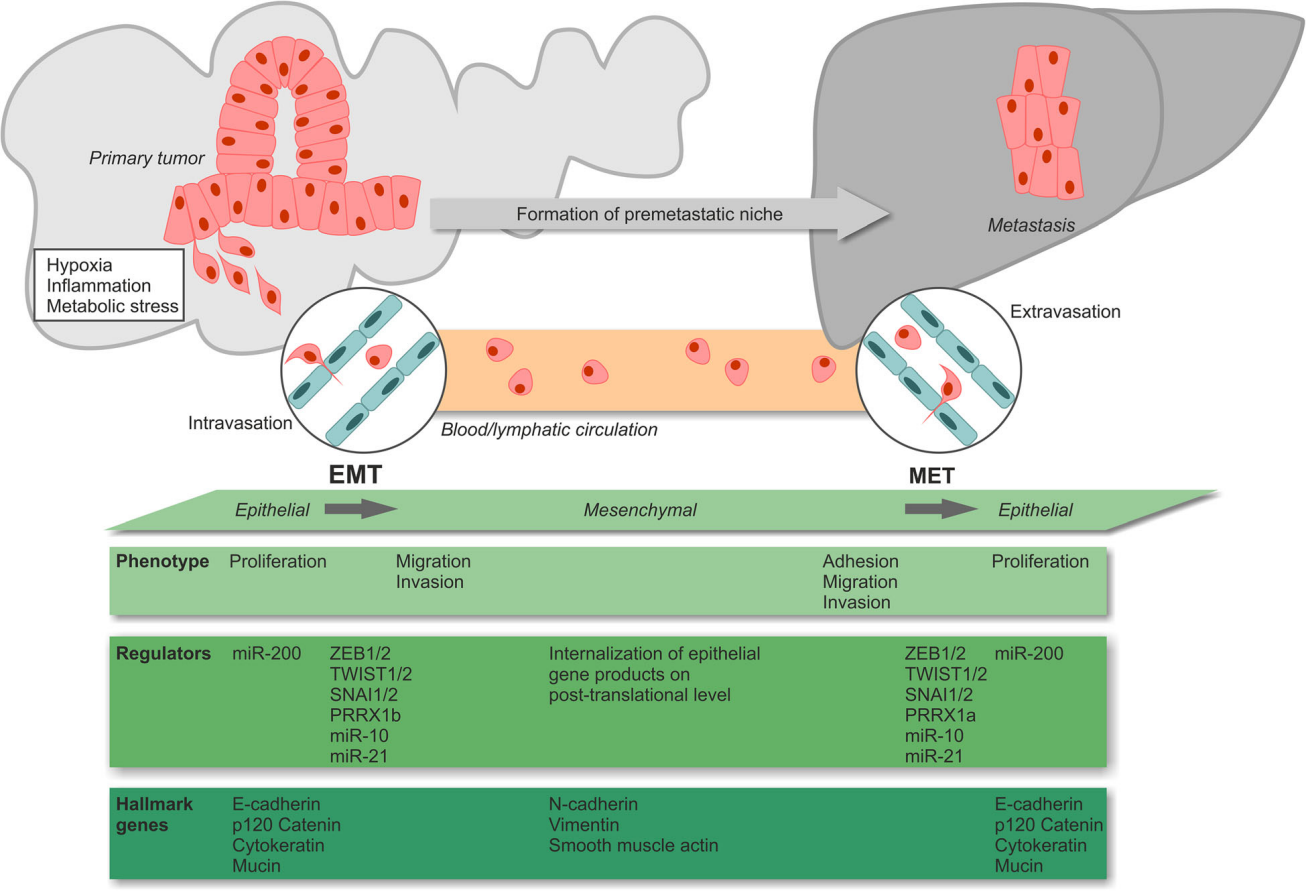
PDAC epithelial-mesenchymal transition and metastasis formation.

Another feature of PDAC is its early progression to metastatic disease [1]. In advanced stages, patients show invasion of the (retro)peritoneum, the liver, and other gastrointestinal organs, as well as – in some cases – the vascular and/or the nervous system [95]. The key drivers of PDAC metastasis formation are still poorly understood, especially since the genetic composition of most metastases is closely resembling the one of the corresponding primary tumors [96–98]. Nevertheless, metastasis formation appears to be a clonal process, since primary PDAC tumors are composed of different subclones with individual metastatic potential, and most of the metastases show high levels of clonality, indicating that they initially evolved from one or only a few disseminated tumor cells [96, 98]. Mechanistic studies with genetically traceable mouse models identified a crucial involvement of epithelial-to-mesenchymal transition (EMT) explaining also why the quasi-mesenchymal PDAC subtype as characterized by stronger expression of mesenchymal genes may be associated with poorer prognosis due to accelerated metastasis formation [61, 62, 99] (Fig. 4). EMT so far has been considered to be orchestrated by a complex network of transcription factors which repress epithelial gene expression and/or induce mesenchymal gene expression, including *twist-related protein 1* and *2 (TWIST1/2)*, *snail family zinc finger protein SNAI1* and *2 (SNAI1/2)*, *zinc finger E-box-binding homeobox 1* and *2 (ZEB1/2)*, and *paired mesoderm homeobox protein 1* (*PRRX1a/b)* [100, 101]. Especially the EMT activator *ZEB1* has been assigned a central role for tumor cell plasticity and metastasis formation in murine PDAC models [102]. miRNAs, particularly miR-10, miR-21 and members of the miR-200 family, constitute another regulatory level of EMT and are closely interlinked with the EMT transcription factors via diverse feedback and feedforward circuits [103, 104]. Recently, a novel, partial program of EMT has been described which is driven by post-translational internalization of epithelial proteins resulting in cluster-like rather than single-cell dissemination [105].

Several parameters of the tumor micro- and macroenvironment are known to influence EMT regulation. Amongst those, hypoxia, inflammation, and metabolic stress appear to be of special importance [100]. Interestingly, high blood glucose concentration, a crucial characteristic of diabetes, has also been shown to facilitate EMT and metastasis formation [7], thus linking a documented risk factor to a relevant tumorbiological process. In order to colonize foreign tissues circulating PDAC cells must undergo a reverse form of EMT (MET) and re-acquire the epithelial state [106, 107]. Morphologically and mechanistically, MET displays many features of EMT in an inverse manner. However, the details of this process as well as its master regulators are still being investigated.

EMT/MET phenomena seem to be crucial elements in the process of metastasis formation, yet gene expression profiling and epigenomic comparisons between primary tumor cells and metastatic cells also disclosed an involvement of other mechanisms, such as rewiring of the carbohydrate metabolism, e.g. in the oxidative branch of the pentose phosphate pathway, as well as shifts in energy consumption [58, 108, 109]. Further studies revealed a (re-)activation of embryonic programs and/or elevated expression levels of cancer stem cell markers, including *forkhead box protein A1 (FOXA1)*, *aldehyde dehydrogenase 1 (ALDH1), ATP-binding cassette sub-family G member 2 (ABCG2),* and *hepatocyte growth factor receptor (c-Met)*, in metastatic PDAC cells, suggesting a close relationship between retrograde developmental transition, cancer cell stemness and biological features of metastasis formation [57, 110]. Finally, the primary tumor appears to condition the future target organ of metastasis by releasing soluble factors and/or exosomes, thus generating a pre-metastatic niche – even in the status of a premalignant lesion [111]. Key players in this regard have been identified to be *tissue inhibitor of metalloproteinases 1 (TIMP-1)* and *macrophage migration inhibitory factor (MIF)* [112, 113].

#### Therapy resistance

A signature hallmark of PDAC is its high degree of resistance against virtually any kind of therapy [114–116]. Accordingly, overcoming treatment resistance will be essential in order to improve the overall prognosis of PDAC.

The therapeutic success of current first-line chemotherapy involving cytidine analogues, the poly-chemotherapeutic protocol FOLFIRINOX, or gemcitabine plus nab-paclitaxel, respectively, is strongly limited by intrinsic and/or acquired chemoresistance, and the underlying mechanisms are only poorly understood [21, 115]. Several predictive biomarkers have been identified, e.g. increased expression of *ribonucleotide reductase catalytic subunits M1/2 (RRM1/2)*, an enzyme catalyzing the reduction of ribonucleotides, or *human equilibrative nucleoside transporter 1 (hENT1),* a transmembrane protein which imports nucleosides into the cytosol [117, 118]. In preclinical studies, it was observed that elevated expression levels of *RRM1* indeed mediate resistance of PDAC cells to gemcitabine [117–119], yet no association between *RRM1* expression and OS was detected in clinical analyses [120]. Similar examples are given by *integrin-linked kinase* (*ILK*) [121] and hypoxia-inducible, pro-apoptotic factor *BCL2/adenovirus E1B 19 kDa protein-interacting protein 3 (BNIP3)* [122]. Furthermore, cells of the microenvironment limit the efficacy of gemcitabine treatment. Recent data show that CAFs contribute to gemcitabine failure by metabolizing gemcitabine to the active metabolite 2′,2′-difluorodeoxycytidine-5′-triphosphate (dFdCTP). However, since dFdCTP cannot cross cell membranes, this process scavenges gemcitabine and reduces the effective concentration of the active metabolite in the tumor cells [123]. In case of FOLFIRINOX treatment, increased expression of *thymidylate synthase* (*TS*) and the 5-FU-catabolizing enzyme *dihydropyrimidine dehydrogenase* (*DPD*) were shown to contribute to therapy resistance, both in preclinical models and in retrospective clinical analyses [119, 124]. However, despite all these efforts, biomarker-based, individualized chemotherapy protocols are far from being clinical standard. This is predominantly due to a lack of prospective validation studies, let alone randomized controlled trials.

PDAC tumors also exhibit a high degree of radioresistance often resulting in tumor progression even during therapy [125]. As in case of chemoresistance, the responsible mechanisms appear to be multifactorial. From a biophysical point of view, the hypoxic PDAC microenvironment reduces the biological effectiveness of photon irradiation by 2-3 fold as compared to well-oxygenated tissues and, thus, attenuates its therapeutic efficacy [126, 127]. Additionally, several studies revealed an overexpression of key regulators of the DNA damage response, e.g. *RAD51*, in PDAC which contribute to accelerated repair of radiation-induced DNA damage [128, 129]. Other studies provided evidence for an implication of *Integrin-* or *SMAD* signaling in PDAC radioresistance [130–132]. Finally, increased recruitment of monocytes upon irradiation stimulating tumor cell proliferation and neovascularization in response to therapy have been discussed [133]. In order to counteract PDAC radioresistance, several approaches focused on adjusting radiotherapeutic protocols. As such, radiotherapy meanwhile is frequently combined with concomitant chemotherapy (radiochemotherapy), using gemcitabine, 5-FU, or capecitabine as radiosensitizing agents [134, 135]. Additionally, stereotactic irradiation regimens with higher single doses, including SBRT and ablative body radiotherapy, are increasingly being employed aiming at the delivery of higher biologically active doses to the tumor [26, 31, 136]. However, therapeutic success is still rather limited, and future attempts should evaluate the clinical potential of biologically and/or immunologically optimized radiochemotherapy strategies.

### Novel approaches of mechanism-based, molecularly targeted therapies

#### Biologically targeted therapies (1,363 words)

Since less than 20% of all PDAC patients exhibit surgically resectable disease at time of presentation, systemic chemotherapy is currently the most frequently applied treatment option [21]. Albeit the development of novel poly-chemotherapy protocols, the overall prognosis, and survival rate of PDAC patients still remain poor. Hence, there is a strong demand for novel, biologically motivated treatment strategies with higher specificity for PDAC-relevant, tumor-driving targets. The genomic landscape of PDAC is dominated by a handful of signature genes which are affected by aberrations and mutations at high frequencies: *KRAS*, *CDKN2A*, *TP53,* and *SMAD4* [49, 51]*.* All of these genes are still basically considered to be undruggable, although agents targeting mutant *TP53* have been developed, and attempts to pharmacologically manipulate *RAS* function are constantly increasing [137, 138]. So far, substances targeting downstream effectors of these major PDAC drivers or other regulators which are also frequently altered, including *BRAF*, *ERK, PI3K/AKT*, and *mTOR,* are in the focus of investigation.

The *mitogen-activated protein kinase* (*MAPK*) signaling cascade offers promising perspectives in this regard, because PDAC cells are known to depend on *MAPK* signaling, both in terms of progression and metastasis formation [139, 140]. The most apical possibility to interfere with *MAPK* signaling is targeting the *epidermal growth factor receptor* (*EGFR*). However, a phase III trial evaluating the efficacy of anti-*EGFR* treatment with cetuximab in addition to gemcitabine-based chemotherapy showed no significant improvement in clinical outcome [141]. Recent data attributed this to a compensatory activation of *Integrin β1* signaling [142]. Downstream of *EGFR, KRAS* constitutes a near-perfect target for PDAC treatment as revealed by preclinical RNA interference experiments [143]. However, clinical RNA interference is challenging, and no reliable *KRAS* inhibitors have been described so far [144]. Nevertheless, pharmacological disruption of the interaction between *KRAS* and phosphodiesterase *PDEδ* was shown to efficiently suppress PDAC progression *in vitro* and *in vivo* [145]. The only targeting approach for *MAPK* signaling that has entered the clinical routine thus far is the combination of gemcitabine and the *EGFR*-specific tyrosine kinase inhibitor erlotinib [146]. Although *EGFR* is considered to be its only target, erlotinib was reported to be similarly effective in tumors with wildtype or hyperactive mutants of *KRAS*, respectively [147]. This implies that either inhibition of tyrosine kinases other than *EGFR* or feedback regulatory mechanisms between hyperactivated *KRAS* and *EGFR* may be involved, respectively [148–151]. Sunitinib, a tyrosine kinase inhibitor that does not target *EGFR,* failed to show similar performance when combined with gemcitabine [152], and preclinical data support the notion that indeed inhibition of gemcitabine-induced *MAPK* signaling by erlotinib accounts for the observed clinical benefits [153]. Several other inhibitors of *MAPK* signaling, including inhibitors of *EGFR, MEK*, *ERK,* and corresponding protein phosphatases, have shown convincing performance in preclinical studies [154–156], but their potential for clinical implementation remains to be examined, as for instance in ACCEPT, a randomized phase II trial combining gemcitabine with the *EGFR* inhibitor afatinib (NCT01728818).

Single-drug treatments – most likely – will not be sufficient to improve the therapeutic outcome of PDAC [157]. Instead, dual or even multiple targeting strategies appear to be required in order to achieve significant advances. One example is the concomitant inhibition of *MAPK* and *PI3K/AKT* signaling. Preclinical data revealed that inhibition of *MAPK* signaling results in potent compensatory activation of *PI3K/AKT* signaling and *vice versa,* each being of importance for PDAC progression [158, 159]. Indeed, concomitant inhibition of *MAPK* and *PI3K/AKT* signaling did interfere with tumor progression to significantly greater extent than the single-drug treatments in preclinical PDAC models [158, 160]. However, other studies reported only modest effects of combined *MAPK* and *PI3K/AKT* inhibition [161–163], and clinical trialing of this combination failed [164]. One potential explanation could be that inhibitors of different target specificities were employed. A more detailed characterization of the target spectrum of these inhibitors would clarify this and could also help to find new targets for mechanism-based therapies. In this regard, upstream and/or transcriptional regulators of *PI3K* expression, such as *transducin beta-like 1 (TBL1),* may also be of interest as studies in genetic mouse models have identified them as crucial checkpoints in PDAC development and progression [165]. Nevertheless, if this mechanism can be exploited therapeutically remains unclear [166].

The *mammalian target of rapamycin* (*mTOR*) pathway is best known for its functions in cell survival, proliferation, motility, and evasion of apoptosis [167]. In several preclinical studies, *mTOR* inhibitors revealed promising results [168–171], but it was also reported that inhibition of *mTOR* stimulates feedback activation mechanisms involving *MEK/ERK* or *AKT* signaling, respectively, further emphasizing the need for combinatorial treatment regimens [172–176]. Not surprisingly, multi-pathway inhibition regimens are commonly associated with higher levels of toxicity [177]. This toxicity often interferes with clinical implementation. Nevertheless, clinical trials evaluating *mTOR* inhibition as monotherapy in PDAC altogether failed [178–180], and combined modality approaches of *mTOR* inhibition in conjunction with capecitabine revealed only limited improvements as compared to capecitabine alone [181]. These findings raise the question whether *mTOR* inhibitors, despite their successful clinical implementation for the treatment of neuroendocrine pancreatic tumors, may at all represent a therapeutic alternative for the treatment of PDAC [182], or whether such approaches have been inadequately tested in the clinic.

PDAC is commonly considered a hypovascularized tumor [183], but relevant expression of *vascular endothelial growth factor A* (*VEGF-A*) has been observed [184]. Therefore, the *VEGF-A*-specific antibody bevacizumab was tested in combination with gemcitabine in a randomized phase III trial with locally advanced PDAC but failed to show improved outcome [185]. A possible explanation could be the expression of other *VEGF* isoforms. However, complementary phase III trials which evaluated the *VEGF receptor* tyrosine kinase inhibitor axitinib in combination with gemcitabine, or the combination of bevacizumab, gemcitabine, and erlotinib, respectively, also failed [186, 187]. In summary, these results render therapeutic targeting of angiogenesis a questionable approach for the treatment of PDAC [188].

A subset of PDAC tumors (approximately 15% of all cases) is characterized by mutations in genes that are related to the DNA damage response [54]. Amongst those, PDAC tumors carrying mutations in *BRCA1/2* genes are of highest interest as they are supposed to be defective in homologous recombination DNA damage repair [189]. Accordingly, patients with *BRCA1/2*-mutated tumors were reported to benefit significantly more from platinum-based chemotherapy than patients with *BRCA1/2* wildtype tumors [190, 191]. For *BRCA1/2*-deficient tumors, the inhibition of *Poly-(ADP-ribose)-polymerase* (*PARP*) may be promising, since this enzyme shares an axis of synthetic lethality with *BRCA1/2* [192]. Initial trials examining the therapeutic potential of *PARP* inhibitors in patients with *BRCA1/2*-deficient PDAC reported promising results [193–196]. Currently, the randomized phase III POLO trial is evaluating *PARP* inhibition in patients who received first-line platinum-based chemotherapy, and results are awaited in 2019 (NCT02184195). Beyond *BRCA1/2*, mutations in other genes of the DNA damage response, including *ATM*, may select for PARP inhibitor sensitivity [197].

In addition to the described genetic alterations, PDAC tumors display relevant changes in epigenetic modifications, including DNA methylation, histone post-translational modification, nucleosome remodeling, and regulation by non-coding RNAs [56]. In contrast to genetic alterations, epigenetic modifications are in principle reversible, and it is plausible to assume that pharmacological interference with epigenetic mechanisms underlying PDAC pathology and progression could open new therapeutic perspectives [198]. Preclinical results of epigenetic therapies have so far been promising, PDAC cell plasticity could be reduced, and resistance against standard chemotherapy was attenuated. However, in mono-agent settings, epigenetic therapeutics did not provide any measurable benefits, demanding for combined modality settings, e.g. in conjunction with chemotherapy or in form of multi-agent combinations, such as combined inhibition of *bromodomain and extra-terminal motif (BET)* proteins and *histone deacetylases (HDACs)* [199]. Currently, various phase I/II trials are ongoing which will determine the clinical perspectives of such approaches. Despite all efforts, individualized, mechanism-based treatment strategies for PDAC are still far from being clinical standard [200].

Therapeutic targeting of hypoxia and metastasis formation appears to be very attractive in the PDAC context, since hypoxia is a principal determinant of therapy resistance and metastasis formation, and metastases are the major cause of death [20, 74]. Regardless of all preclinical efforts [201], however, no therapeutic strategy could so far be established. Sort of alternatively, efforts to (re-)activate the immune system in order to detect and combat macro- and micro-metastases have been undertaken and will be discussed in the following.

#### Immunotherapy

Immunotherapy implementing immune checkpoint inhibitors has revolutionized cancer treatment in the last years [202]. Therapeutic antibodies targeting *cytotoxic T-lymphocyte-associated protein 4* (*CTLA-4)* or the axis of *programmed cell death protein 1 (PD-1)* and its corresponding ligand *PD-L1* have shown compelling results in several different cancer types, including metastasized melanoma and lung cancer [36, 203]. Hence, immune checkpoint inhibition was also tested in PDAC [35, 39], but compared to melanoma and lung cancer, considerably smaller numbers of patients (approximately 2%) exhibited clinical benefits [40, 204]. Consistently, the responding tumors showed high levels of microsatellite instability, providing a mechanistic explanation as well as a potential future stratification marker, since microsatellite instability is known to increase the number of tumor-associated neo-antigens [205].

A major determinant of the immunotherapeutic success are tumor-specific T cells and their (re-)activation. Although their numbers have been described to be rather low in PDAC patients [90], recent data suggest that the tumor-reactive T-cell repertoire is similar to the one found in melanoma where T cell-based therapies meanwhile have relevant therapeutic impact [91]. Further studies showed that neo-antigen quality rather than quantity, and strong intra-tumoral CD8^+^ T cell infiltration are associated with prolonged survival, indicating that the stimulation of anti-tumor T cell responses can indeed be a promising strategy for the treatment of PDAC [60, 206, 207]. Along these lines, different vaccination strategies employing various kinds of antigens have already been tested [208–210]. The Algenpantucel-L vaccine consisting of irradiated, allogeneic pancreatic tumor cells stably expressing *alpha-1,3-galactosyltransferase 2* (*A3GALT2*), a glycosylating enzyme that mainly targets lipids and extracellular proteins, turned out to be the most promising candidate for a PDAC-targeting vaccine [209]. However, this vaccine failed to improve treatment efficacy when being tested in a randomized phase III trial combined with the standard of care [211]. Other antigens that were examined include peptides derived from human *telomerase 1* (*TERT1*) and GVAX, a vaccine comprised of autologous or allogeneic tumor cells expressing the dendritic cell-stimulating cytokine *GM-CSF* [212, 213]. Unfortunately, none of these vaccines achieved convincing clinical results. In principle, common PDAC driver mutations, such as *KRAS*^*G12D*^, can harbor tumor-specific, T cell epitopes [214]. An ongoing phase II trial first predicts such neo-antigens using exome-sequencing of tumor biopsies, followed by production of personalized dendritic cell vaccines loaded with the respective epitopes (NCT03300843) [215]. Whether this strategy turns out to be successful needs to be awaited. Overall, several vaccination approaches could successfully elicit measurable anti-tumor T cell responses, yet so far none of these strategies resulted in clear clinical benefits [216].

Antigen-independent immunostimulatory therapies aim at the activation of antigen-presenting cells. Diverse receptor-ligand-axes have been explored in this regard. As such, treatment with agonistic anti-CD40 antibodies is well known to activate antigen-presenting cells and to polarize macrophages towards the pro-inflammatory M1-like state [217, 218]. However, clinical evaluation of this strategy in PDAC patients disclosed only short-term responses, and no long-term anti-tumor immunity was observed [219]. Nevertheless, CD40 stimulation in combination with chemotherapy and immune checkpoint blockade is currently under clinical investigation in a phase I/II trial (NCT03214250). Complementary approaches to achieve activation of antigen-presenting cells involve ligand-dependent stimulation of *pattern recognition receptors (PRRs)* [220]. Indeed, agonists of *toll-like receptors (TLRs)*, *RIG-I-like helicases (RLHs),* and the *stimulator of interferon genes* (*STING*) revealed encouraging results in preclinical PDAC models [221–223], but their clinical potential remains to be elucidated.

Bypassing the *in situ* steps of T cell priming by antigen-presenting cells, adoptive transfer of T cells carrying chimeric antigen receptors (CARs) has proven powerful clinical performance in B-cell malignancies [224]. CAR T cells recognize specific cancer cell surface antigens through a single-chain variable fragment (scFv) whose ligation stimulates T cell activation via the intracellular domains of the CAR construct, resulting in efficient T cell-mediated killing of the target cell [225]. PDAC exhibits several tumor-specific antigens, such as *carcinoembryonic antigen (CEA)*, *mesothelin (MSLN)*, and *mucin 1 (MUC1)*, which are promising determinants for CAR T cell therapy [226, 227]. However, for solid cancer entities, intra-tumoral recruitment and trafficking of CAR T cells as well as the commonly observed immunosuppressive tumor microenvironment appear to be major challenges. Intelligent combinations, thus, are needed in order to overcome these obstacles.

A cardinal feature of the immunosuppressive PDAC microenvironment is its massive stromal content and the excessive deposition of extracellular matrix, including hyaluronan [72]. Early phase clinical trials combining recombinant human *hyaluronidase 20* (*rHuPH20*) with gemcitabine and nab-paclitaxel revealed promising results, particularly in those patients whose tumors were characterized by high levels of hyaluronan [228]. Reporting of the HALO-109-301 phase III trial (NCT02715804) is awaited in order to fully assess the clinical performance of this approach [229]. Inhibition of *FAK1*, a tyrosine kinase involved in the process of CAF generation, constitutes another approach to interfere with stromal function in PDAC, and pharmacological *FAK1* inhibition eventually rendered preclinical PDAC model systems more susceptible to T cell immunotherapy and immune checkpoint inhibition [73]. Other studies showed that genetic ablation or inhibition of *FAK1* also increases PDAC responsiveness to gemcitabine and nab-paclitaxel [230, 231]. In rather strong contrast, genetic deletion of stromal myofibroblasts in PDAC mouse models led to disease exacerbation and diminished animal survival due to enhanced regulatory T cell-mediated immunosuppression, clearly calling for caution when targeting components of PDAC stroma [78].

On a cellular level, massive infiltration by myeloid cells, such as MDSCs, and resulting exclusion of CD8^+^ T cells are major hallmarks of the immunosuppressive PDAC microenvironment [86, 232]. Several myeloid cell-targeting approaches have been investigated in recent years in order to overcome these mechanisms of immunosuppression [82, 233, 234]. *Chemokine receptor 2 (CCR2)*, for instance, is known to contribute to the infiltration of pancreatic tumors by monocytes and macrophages, and this is associated with reduced patient survival and poor outcome [235]. Strikingly, the combination of *CCR2* blockade and gemcitabine/nab-paclitaxel chemotherapy showed promising results in phase I trials [85, 236]. However, the follow-up phase Ib/II trial (NCT02732938) was discontinued due to strategic considerations, and instead phase I/II trials with combined modality approaches of CCR2 blockade in conjunction with pre-operative SBRT and immune checkpoint inhibition were recently initiated (NCT03778879, NCT03767582). Another target that regulates the function of macrophages and MDSCs in PDAC is *M-CSF*. Preclinical data suggest that *M-CSF* blockade can indeed reprogram macrophages and thus, synergize with immune checkpoint inhibition, but the clinical potential of this strategy remains to be examined [237].

In summary, (re-)activating anti-PDAC immunity in order to improve the overall clinical outcome appears clearly more challenging than extrapolated experiences from other cancer entities have suggested. Probably the most promising strategies would incorporate combinations of different immunotherapeutic approaches and/or combinations with other (classical) treatment modalities, such as chemotherapy and/or radiotherapy [238].

### Combined modality treatment approaches encompassing radio(chemo)therapy

**Fig. 5:**
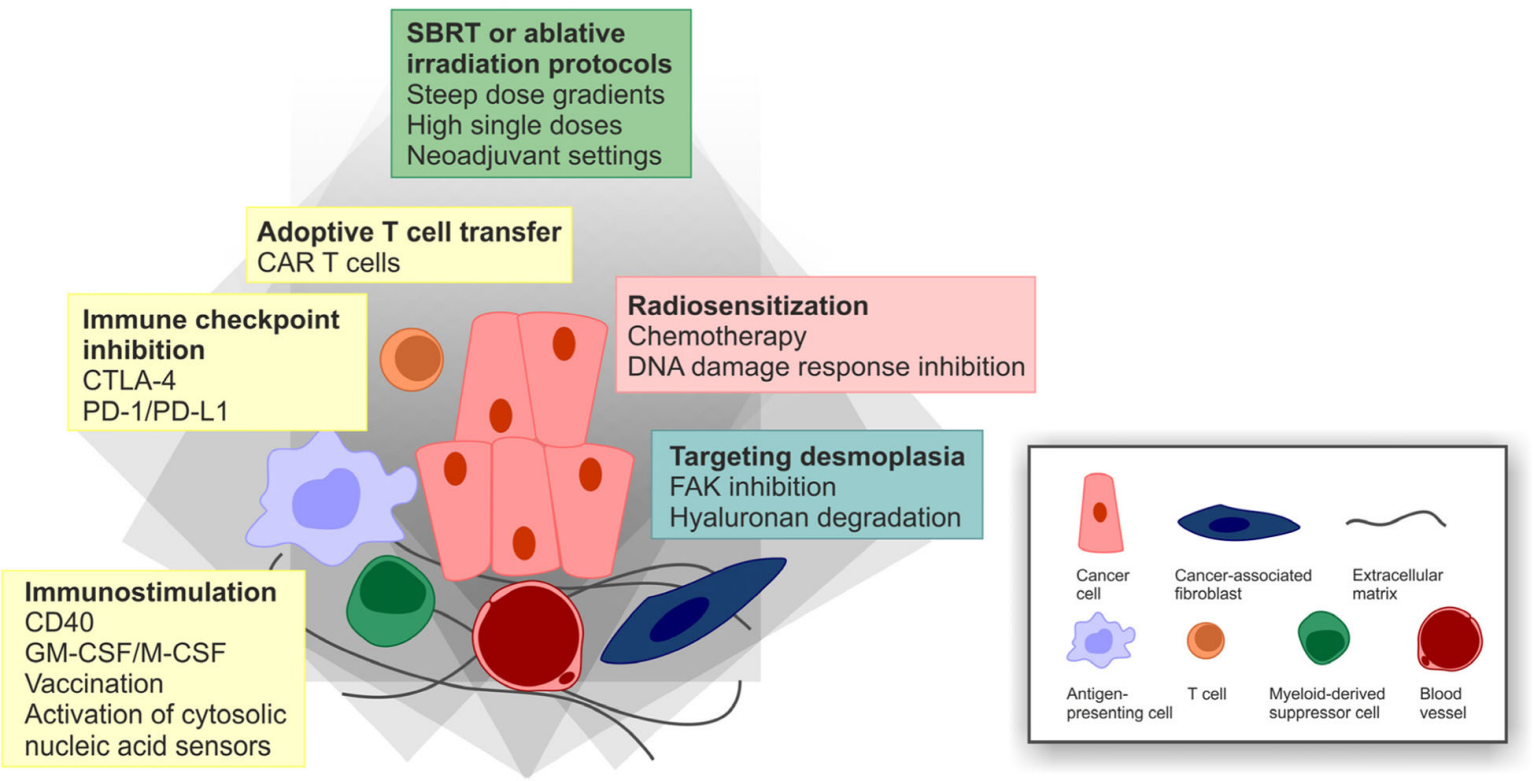
Combined modality perspectives for the treatment of PDAC.

In order to improve the efficacy and the outcome of clinical PDAC treatment, it will be inevitable to develop novel treatment strategies which combine different therapeutic modalities aiming at achieving synergism [239]. The rationale for such approaches is to outcompete therapy resistance, but their development remains challenging as combined modality treatments are frequently associated with higher toxicity levels [240]. We already discussed several combined modality attempts involving different chemotherapeutics, either with each other or with novel, molecularly targeted inhibitors. At this point, we want to concentrate on combinatorial approaches involving radiotherapy (Fig. 5).

Radiotherapy has rather infrequently been used for the treatment of PDAC. Nevertheless, there have been approaches to improve the efficacy of radiotherapy in PDAC. One obvious strategy is to combine radiotherapy with radiosensitizing agents which either can be classical chemotherapeutic drugs, such as gemcitabine or 5-FU, or – as has been reported more recently – molecularly designed inhibitors that target specific proteins and/or structures involved in PDAC radioresistance [28, 125]. The *MAPK* pathway is a very attractive target [140], and preclinical data derived from different PDAC mouse models showed that interference with *MAPK* signaling by cetuximab treatment can indeed increase the efficacy of radiochemotherapy [241, 242]. Encouraged by these observations, several clinical trials were initiated, yet with only modest results [243–246]. The major reason was the persistently high rate of distant failure due to metastasis formation, rather than poor local control [244, 246].

Pharmacological intervention with the *PI3K/AKT* and the *mTOR* pathway has also been examined with regards to its radiosensitizing potential. Several preclinical studies obtained basically positive results [247–253]. However, due to very unfavorable pharmaceutical properties of the employed substances, e.g. elevated toxicity levels and crossover inhibition, none of these approaches have entered the clinic thus far.

A very direct approach of radiosensitization is the employment of molecularly designed drugs which target components of the DNA damage response, specifically the upstream kinases *ATM*, *ATR*, *CHK1/2,* and *DNA-PK* [254–256]. Several of these inhibitors displayed convincing synergism with ionizing irradiation or DNA-damaging chemotherapy in preclinical PDAC model systems [257–263], but the transferability into the clinic remains to be investigated – particularly in view of local control versus distant failure. *PARP* is another example for a DNA damage response regulator that can be targeted by highly refined inhibitors, and preclinical data suggest that *PARP* inhibition indeed can radiosensitize PDAC cells [264]. However, since *PARP* is known to share synthetic lethality with *BRCA1/2* [192], *PARP* inhibition may turn out to be only effective in *BRCA1/2* deficient tumors [265]. This is a general lesson that has been learned in the era of molecularly targeted therapy: Molecularly designed therapy requires upfront molecular diagnostics and proper patient stratification, since otherwise promising agents are prone to fail if they are trialed in the wrong subgroups of patients.

Apart from its potential to induce tumor cell death, radiotherapy is known to recondition the tumor microenvironment and to stimulate systemic anti-tumor immune responses – a phenomenon summarized as abscopal effects of radiotherapy [266–268]. However, in the monotherapy setting, radiation is often not sufficient to break the immunosuppressive milieu of established tumors, and combinations with immunostimulating agents are required. As an example, radiotherapy plus *GM-CSF*, a potent stimulator of antigen-presenting cell maturation, produced objective abscopal responses in a subset of patients with different metastatic tumors [269], and a recent case report showed similar effects in a patient with metastatic pancreatic cancer [270]. In preclinical model systems, PDAC tumors have been reported to regress convincingly upon immunotherapeutic targeting of *CCL2* or *PD-L1* in combination with radiotherapy via a reduction of intra-tumoral immunosuppressive myeloid cells and enhanced recruitment of tumor-specific T cells [133, 271], and the clinical performance of this approach will be investigated (NCT03778879, NCT03767582). Similarly, radiotherapy has been described to reprogram tumor-infiltrating macrophages towards an M1-like phenotype and to favor intra-tumoral recruitment of adoptively transferred T cells in a mouse model of neuroendocrine pancreatic cancer [272]. These observations were confirmed by pilot data from patients with advanced PDAC stages undergoing neoadjuvant irradiation prior to tumor resection revealing 3- to 5-fold increases in intra-epithelial CD4^+^ and CD8^+^ T cells as compared to non-irradiated control patients [272, 273]. If these findings may also be transferred to combinations with PDAC-specific CAR T cells remains to be examined. On a mechanistic level, cytosolic DNA-sensing upon irradiation-induced DNA damage and type I interferon signaling appear to be involved in the immunostimulating effects of radiotherapy [274, 275]. Accordingly, artificial activation of cytosolic DNA sensors, such as *STING*, was shown to increase the efficacy of radiotherapy by enhancing CD8^+^ T cell responses – at least in preclinical PDAC models [276].

From clinical experiences with other cancer entities it is becoming increasingly evident that the combination of radiotherapy and immunotherapy requires very careful considerations regarding timing, dosing, and treatment sequence in order to achieve the best outcome [266]. This may be of particular interest for PDAC with its highly challenging immunosuppressive microenvironment. In brief, higher single doses of radiotherapy, e.g. SBRT or ablative protocols, applied in neoadjuvant settings appear to be beneficial, and immunotherapy needs to be started before or with the first irradiation fraction, respectively [266]. However, the optimal treatment regimen and the best combination of agents for PDAC remain unclear as well as the impact of additional chemotherapy and other factors, such as type II diabetes and/or obesity. A pilot study addressing some of these combinatorial issues added radiotherapy to *CD40*-dependent immunostimulation plus anti-*CTLA-4*/anti-*PD-1*-mediated immune checkpoint blockade in genetically engineered PDAC mouse models and utilized machine learning algorithms to extract signature patterns for each therapeutic component [277]. Along these lines, more in depth-analyses are needed in order to fully exploit the synergism between radiotherapy and immunotherapy. Nevertheless, several clinical phase I/II trials combining radiotherapy with different immunotherapeutic approaches have been initiated for advanced PDAC, and first results are awaited [278] (NCT02648282, NCT03161379, NCT03767582, NCT03563248).

## Conclusions

PDAC represents a cancer entity of extraordinarily high malignancy, particularly poor prognosis, and constantly increasing patient numbers. Its aggressive biology and the fact that most patients present in advanced or disseminated stages of disease render the development of novel PDAC treatment strategies one of the superordinate challenges in current oncological research. Results of the last 20 years have led to the establishment of a detailed multi-step model of PDAC development and progression. Although this has unquestionably reformed our understanding of PDAC as a disease, none of these findings could be successfully translated into a therapeutic breakthrough so far. It is becoming increasingly evident that the clinical performance of single-agent therapies lags behind the original expectations, and instead intelligent combinations appear to be required. In this regard, radiotherapeutic protocols, and particularly modern radiation techniques with high conformality and steep dose gradients, represent attractive partners both for biologically motivated as well as for immunotherapeutic strategies. Importantly, however, this will require in-depth optimization of timing, dosing, and treatment sequences, as well as careful upfront patient stratification. Otherwise *per se* promising combinations run the risk of failing prematurely.

## Data Availability

Data sharing not applicable to this article as no datasets were generated or analyzed during the current study.

## Abbreviations

5-FU: 5-Fluorouracil
*A3GALT2*: Alpha-1,3-galactosyltransferase 2
*ABCG2*: ATP-binding cassette sub-family G member 2
ADM: Acinar-to-ductal metaplasia
*AKT*: RAC-beta serine/threonine-protein kinase
*ALDH1*: Aldehyde dehydrogenase 1
*ATM*: Ataxia telangiectasia mutated protein serine/threonine kinase
*ATR*: ATM- and Rad3-related kinase
*BET*: Bromodomain and extra-terminal motif
*BNIP3*: BCL2/adenovirus E1B 19 kDa protein-interacting protein 3
*BRAF*: v-Raf murine sarcoma viral oncogene homolog B
*BRCA1/2*: Breast cancer early onset 1/2
CAF: Cancer-associated fibroblast
CAR T cell: Chimeric antigen receptor T cell
*CCL-2*: CC-chemokine ligand 2
CD: Cluster of differentiation
*CDKN2A*: Cyclin-dependent kinase inhibitor 2A
*CEA*: Carcinoembryonic antigen
*CHK1/2*: Checkpoint kinase 1/2
*c-Met*: Hepatocyte growth factor receptor
*CTLA-4*: Cytotoxic T lymphocyte-associated protein 4
*CXCL-1*: CXC-chemokine ligand 1
*CXCR2*: CXC-chemokine receptor 2
dFdCTP: 2′,2′-difluorodeoxycytidine-5′-triphosphate
*DNA-PK*: DNA-dependent protein kinase
*DPD*: Dihydropyrimidine dehydrogenase
*EGFR*: Epidermal growth factor receptor
EMT: Epithelial-to-mesenchymal transition
*ERK*: Extracellular signal-regulated kinase
*FAK1*: Focal adhesion kinase 1
FOLFIRINOX: Poly-chemotherapeutic regimen composed of folinic acid, 5-FU, irinotecan, and oxaliplatin
*FOXA1*: Forkhead box protein A1
*GM-CSF*: Granulocyte macrophage stimulating factor
*HDAC*: Histone deacetylases
*hENT1*: Human equilibrative nucleoside transporter 1
*HNF1A*: Hepatocyte nuclear factor 1A
IGRT: Image-guided radiotherapy
*IL*: Interleukin
*ILK*: Integrin-linked kinase
*KRAS*: Proto-oncogene from Kirsten rat sarcoma virus
*KRT81*: Cytokeratin-81
*MAPK*: Mitogen-activated protein kinase
*M-CSF*: Macrophage colony stimulating factor
MDSC: Myeloid-derived suppressor cell
*MEK*: Mitogen-activated protein kinase kinase
MET: Mesenchymal-to-epithelial transition
*MIF*: Macrophage migration inhibitory factor
*MSLN*: Mesothelin
*mTOR*: Mammalian target of rapamycin
*MUC1*: Mucin I
NCCN: National Comprehensive Cancer Network
OS: Overall survival
*PALB2*: Partner and localizer of BRCA2
PAnIN: Pancreatic intra-epithelial neoplasias
*PARP1/2*: Poly-(ADP-ribose)-polymerase 1/2
*PD-1*: Programmed cell death 1
PDAC: Pancreatic ductal adenocarcinoma
*PDEδ*: Photoreceptor cGMP phosphodiesterase δ subunit
*PD-L1*: Programmed cell death ligand 1
*PI3K*: Phosphoinositide 3-kinase
*PRR*: Pattern recognition receptor
*PRXX1a/b*: Paired mesoderm homeobox protein 1a/b
rHuPH20: Recombinant human hyaluronidase 20
*RIG-I*: Retinoic acid inducible gene I
*RLH*: RIG-I-like helicases
*RRM1/2*: Ribonucleotide reductase catalytic subunits M1/2
SBRT: Stereotactic body radiotherapy
scFv: single-chain variable fragment
*SMAD4*: Mothers against decapentaplegic homologue 4
*SNAI1/2*: Snail family zinc finger protein 1/2
*STING*: Stimulator of interferon genes
*TBL1*: Transducin beta-like 1
*TERT1*: Telomerase reverse transcriptase 1
*TGF-β*: Transforming growth factor β
*TIMP-1*: Tissue inhibitor of metalloproteinases 1
*TLR*: Toll-like receptor
*TP53*: Tumor protein 53
*TS*: Thymidylate synthase
*TWIST1/2*: Twist-related proteins 1/2
*VEGF-A*: Vascular endothelial growth factor A
*ZEB1/2*: Zinc finger E-box-binding homeobox 1/2
